# The role of an anti-reflux diet in the treatment of chronic cough caused by laryngopharyngeal reflux

**DOI:** 10.1007/s00405-025-09258-3

**Published:** 2025-02-24

**Authors:** Viktória Hránková, Tomáš Balner, Adéla Kondé, Patrícia Gubová, Karol Zeleník, Pavel Komínek, Lucia Staníková

**Affiliations:** 1 Department of Otorhinolaryngology and Head and Neck Surgery, University Hospital of Ostrava, Ostrava, Czech Republic; 2 Department of Allergology and Clinical Immunology, University Hospital of Ostrava, Ostrava, Czech Republic; 3 Department of Craniofacial Surgery, Faculty of Medicine, University of Ostrava, Ostrava, Czech Republic; 4 Department of Biomedical Sciences, Faculty of Medicine, University of Ostrava, Ostrava, Czech Republic; 5 Department of Applied Mathematics, Faculty of Electrical Engineering and Computer Science, VSB– Technical University of Ostrava, Ostrava, Czech Republic; 6 Department of Deputy Director for Science, Research and Education, University Hospital Ostrava, Ostrava, Czech Republic

**Keywords:** Laryngopharyngeal reflux, Chronic cough, Diet, Proximal acid exposure time, Bronchial asthma, Proton pump inhibitors

## Abstract

**Purpose:**

To evaluate the role of an anti-reflux diet in the treatment of patients with chronic cough caused by laryngopharyngeal reflux (LPR).

**Methods:**

This prospective observational study included patients with chronic cough (lasting over 3 months) and laryngopharyngeal reflux (LPR) confirmed by hypopharyngeal-esophageal 24-h multichannel intraluminal impedance-pH monitoring (HEMII-pH), according to Dubai criteria. Participants were categorized based on cough severity using a visual analog scale (VAS) from 1 to 10. A VAS < 5 was considered to indicate mild cough, whereas a VAS ≥ 5 were considered to indicate severe cough. Patients with mild cough were treated by anti-reflux diet only, while those with severe cough received additional treatment with proton pump inhibitors (PPIs) and alginates. After 3 months, treatment effectiveness was evaluated by assessing the reduction in cough severity.

**Results:**

In patients with mild cough, anti-reflux diet alone proved to be effective, yielding improvement in 83.3% of cases. Among patients with severe cough, a combination of anti-reflux diet, proton pump inhibitors (PPIs), and alginates proved was effective in 81.8% of cases.

**Conclusion:**

Diet alone is an effective and sufficient treatment for mild chronic cough in patients with LPR. For patients with severe chronic cough with LPT, combined anti-reflux measures are effective.

## Introduction

Laryngopharyngeal reflux (LPR) is a condition in which stomach contents flow back into the larynx and pharynx, causing irritation and various symptoms—including chronic cough, defined as a cough lasting over 3 months. More than 20% of patients with chronic cough have confirmed LPR [1]. Specialists approach LPR treatment in different ways. Although there are several established therapeutic options, it is unclear which treatment approach is most effective with the lowest risk of complications. The choice of treatment largely depends on how LPR manifests, and its severity, in each individual patient. An anti-reflux diet and lifestyle modifications are mainstays of LPR management [2]. Additionally, treatment may include proton pump inhibitors (PPIs), alginates, prokinetics, H_2_ receptor antagonists and, in severe cases, surgical intervention. During the last two decades, PPIs have been widely used as first-line treatment for LPR [3]. However, a recent randomized controlled trial [4] found no significant difference in effectiveness between PPIs and a placebo. Moreover, PPI treatment may raise the risk of severe infections by disrupting gut microbiota and affecting immune function [5].

In the present prospective study, we evaluated the effectiveness of an anti-reflux diet as a standalone treatment for patients with mild LPR-related chronic cough. Additionally, we explored the outcomes of combining an anti-reflux diet with pharmacological treatment in patients with severe LPR-related chronic cough. The aim of this study was to contribute to a better understanding of the role of diet in managing LPR-induced cough, and to provide insights regarding when additional therapies are necessary. To our knowledge, this is first study to evaluate the role of anti-reflux diet in patients with chronic cough caused by LPR.

## Methods

This prospective cohort study was performed at the Department of Otorhinolaryngology and Head and Neck Surgery of the University Hospital Ostrava, from 2021 to 2024. The study was performed in accordance with the Declaration of Helsinki, the requirements of good clinical practice, and all applicable regulatory requirements. Additionally, this study was approved by the institutional review board of University Hospital Ostrava, and registered at ClinicalTrials.gov (NCT04984304). Written informed consent was obtained from all participants before any procedure was initiated.

### Study design

The study inclusion criteria were patients with chronic cough and confirmed LPR. The exclusion criteria were use of angiotensin-converting enzyme inhibitors or sartans; head and neck cancers or previous radiation therapy in these areas; pulmonary cancers; chronic lung diseases, such as chronic obstructive pulmonary disease or sarcoidosis; and chronic rhinosinusitis. Patients with chronic cough (lasting over 3 months) underwent hypopharyngeal-esophageal 24-h multichannel intraluminal impedance-pH monitoring (HEMII-pH) while off anti-reflux therapy. LPR presence was assessed according to the Dubai criteria, with patients having multiple reflux episodes above the upper esophageal sphincter considered to have LPR [6].

Cough severity was evaluated using a visual analog scale (VAS) ranging from 1 to 10. A VAS score of < 5 was considered to indicate mild cough, whereas a VAS score of ≥5 was considered to indicate severe cough. Patients with mild cough were treated with anti-reflux diet only, while those with severe cough received additional treatment with PPIs and alginates, based on the results of HEMII-pH. After 3 months, treatment effectiveness was evaluated by assessing the reduction in cough severity using the VAS. Treatment was considered successful in cases where cough severity was reduced to a VAS of < 2 in patients with an initially mild cough, and to a VAS of < 4 in patients with an initially severe cough.

### Methodology of 24-h multichannel intraluminal impedance-pH (MII-pH) monitoring

LPR was diagnosed using a Digitrapper^®^ pH-Z system, with a single-use VersaFlex^®^ LPR ZNID22 + 8R probe. The impedance probe contains a dual pH channel at 0 cm (distal) and 22 cm (proximal), and 8 impedance rings. Prior to assessment, patients fasted and discontinued PPI use. The catheter was transnasally introduced, with the distal pH channel and 6 impedance rings positioned below the upper esophageal sphincter, and the proximal pH channel and 2 impedance rings placed in the hypopharynx above the upper esophageal sphincter. Correct probe position and electrode placement were confirmed using flexible endoscopy.

The 24-hour recording was initially analyzed electronically using software, and then manually verified by an experienced otorhinolaryngologist. A laryngopharyngeal reflux episode was defined as an episode reaching both proximal hypopharyngeal impedance sensors.

A hypopharyngeal acid event was defined as an event with a pH < 4.0; a hypopharyngeal weakly acid reflux event as an event with a pH between 4.0 and 7.0; and a hypopharyngeal alkaline reflux event as an event with pH > 7.0 [7].

### Reflux symptoms and findings assessment

The subjective symptoms of LPR and their impact on quality of life were assessed using the Reflux Symptom Index (RSI) and Reflux Symptom Score 12 (RSS-12) questionnaires [8]. An RSI score of > 13 and an RSS-12 of > 11 were considered abnormal and suggestive of LPR. Moreover, endoscopic examination of the pharyngeal and laryngeal mucosa was performed, and the results were evaluated using the Reflux Sign Assessment - Short version (RSA) system [9].

### Treatment intervention

Patients with mild cough (VAS 1–4) were treated with dietary and lifestyle measures for 12 weeks. The anti-reflux diet included high-protein, low-fat, alkaline, and low-sugar foods. Patients were provided with a written diet plan, and received a thorough explanation of the principles and importance of diet. Patients were also instructed on lifestyle measures, including stress management, remaining in an upright position for at least 30 min after meals, and consuming small portions. The group of patients with severe cough (VAS 5–10) was also instructed in therapeutic dietary and lifestyle measures, and was additionally prescribed medication for 12 weeks, according to type of LPR: acidic reflux, diet + PPI twice daily + alginate twice daily; mixed reflux, diet + PPI once daily + alginate twice daily; and weakly acidic reflux, diet + alginate 2–3 times daily.

### Statistical methods

Numerical parameters are presented as median and interquartile range (IQR), or minimum and maximum. Categorical variables are presented as absolute frequency and relative frequency (%). The significance of between-group differences was tested using the Mann–Whitney test, the Chi-square test of independence for contingency tables, or the Fisher’s exact test. The significance level was set to 0.05. Statistical analyses were performed using R software, version 4.4.1 (R foundation, Vienna, Austria).

## Results

### Demographic data and history

For this study, 89 patients were recruited, of whom 9 were excluded because they did not complete follow-up. Thus, the study included a total of 80 patients, comprising 22 men (27.5%) were and 58 women (72.5%). Among these 80 patients, 36 were determined to have mild cough, and 44 severe cough. Table 1 summarizes the demographic data and history of patients in both groups. The groups notably differed in gender distribution, with significantly more women in the severe cough group (*p* = 0.021). The mild and severe cough groups did not significantly differ in any other demographic data (age or BMI) or patient history.

**Table 1:** Patients’ demographic information and medical history according to cough severity

	Median (IQR) or *n* (%)		*p*
	Mild cough (*n* = 36)	Severe cough (*n* = 44)	
Age, years	49 (37; 56)	54 (36; 62)	0.529
Body mass index, kg/m^2^	25.6 (23.9; 27.6)	26.1 (23.9; 29.8)	0.764
Sex			**0.021**
Female	21 (58.3)	37 (84.1)	
Male	15 (41.7)	7 (15.9)	
Smoking			0.906
Yes	7 (19.4)	7 (15.9)	
No	29 (80.6)	37 (84.1)	
Alcohol			0.353
Occasionally	20 (55.6)	30 (68.2)	
No	16 (44.4)	14 (31.8)	
Heartburn			0.181
More than once a week	6 (16.7)	11 (25.0)	
Occasionally	13 (36.1)	8 (18.2)	
No	17 (47.2)	25 (56.8)	
Bronchial asthma			0.295
Yes	12 (33.3)	9 (20.5)	
No	24 (66.7)	35 (79.5)	
Another allergy			0.866
Yes	8 (22.2)	8 (18.2)	
No	28 (77.8)	36 (81.8)	
Proton pump inhibitors			0.428
More than once a week	6 (16.7)	9 (20.5)	
Less than once a week	9 (25.0)	6 (13.6)	
No	21 (58.3)	29 (65.9)	

Data are presented as median and interquartile range (IQR) or absolute and relative frequencies (%). The *p*-value was obtained with the Mann-Whitney test or the Chi-square test of independence for contingency tables

### Reflux symptoms, findings, and HEMII-pH results

Table 2 summarizes the comparison of reflux symptoms, findings, and HEMII-pH results. The two groups did not significantly differ in RSI, RSS-12, or RSA-Short version. However, patients with severe cough more frequently exhibited PAET > 1% (*p* = 0.005) and showed significantly higher numbers of total and acid reflux hypopharyngeal episodes (Table 2).

### Improvement after 12 weeks of treatment

In the group of patients with mild cough, after 12 weeks of treatment with diet and lifestyle modifications, 30/36 (83.3%) patients exhibited improvement. In the group of patients with severe chronic cough, after 12 weeks of treatment with a combination of diet, lifestyle modifications, PPIs, and alginate, 36/44 (81.8%) patients exhibited improvement. Overall, treatment was successful in 66/80 (82.5%) patients with chronic cough caused by LPR.

**Table 2:** Patients’ clinical examination, therapy, and improvement according to cough severity

	Median (IQR) or *n* (%)		*p*
	Mild cough (*n* = 36)	Severe cough (*n* = 44)	
RSI	13 (9; 18)	14 (11; 18)	0.545
RSS-12	32 (20; 70)	44 (23; 80)	0.356
RSA Short version	25 (19; 32)	20 (14; 30)	0.084
DeMeester positive			0.102
Yes	18 (50.0)	31 (70.5)	
No	18 (50.0)	13 (29.5)	
PAET (%)	0.6 (0.3; 6.4)	4.2 (0.7; 5.6)	0.237
PAET > 1%	14 (38.9)	32 (72.7)	**0.005**
Number of reflux episodes			
Total	5 (3; 8)	12 (6; 19)	**< 0.001**
Acidic	3 (2; 7)	8 (4; 14)	**< 0.001**
Weakly acidic	1 (0; 3)	3 (0; 5)	0.111
Alkaline	0 (0; 0)	0 (0; 0)	---
Improvement after 12 weeks treatment			> 0.999
Yes	30 (83.3)	36 (81.8)	
No	6 (16.7)	8 (18.2)	

Data presented as median and interquartile range (IQR) or absolute and relative frequencies (%). The *p*-value was obtained with the Mann-Whitney test or the Chi-square test of independence for contingency tables

*PAET* proximal acid exposure time, *RSI* Reflux Symptom Index, *RSS 12* Reflux Symptom Score 12, *RSA Short version* Reflux Sign Assessment

### Sub-analysis of patients with mild cough

Sub-analysis of patients with mild cough (Table 3) revealed that the patients who did not response to diet and lifestyle modifications had significantly lower RSI (*p* = 0.041) and significantly fewer hypopharyngeal reflux episodes on HEMII-pH (*p* = 0.034).

**Table 3:** Comparison of patients with mild cough according to improvement

	Median (Min; Max) or *n* (%)		*p*
	Improvement (*n* = 30)	No improvement (*n* = 6)	
RSI	15 (1; 32)	8 (1; 18)	**0.041**
RSS-12	35 (8; 168)	28 (10; 72)	0.596
RSA Short version	23 (13; 120)	28 (17; 35)	0.328
DeMeester positive			> 0.999
Yes	15 (50.0)	3 (50.0)	
No	15 (50.0)	3 (50.0)	
PAET (%)	0.6 (0.0; 9.8)	3.0 (0.0; 7.3)	0.782
PAET > 1%	11 (36.7)	3 (50.0)	0.878
Number of reflux episodes			
Total	5 (2; 32)	2 (2; 7)	**0.034**
Acidic	3 (0; 23)	2 (0; 7)	0.335
Weakly acidic	2 (0; 9)	0 (0; 2)	0.152
Alkaline	0 (0; 2)	0 (0; 0)	---

Data presented as median and the minimum and maximum or absolute and relative frequencies (%). The *p*-value was obtained with the Mann-Whitney test or the Fisher’s exact test

*PAET* proximal acid exposure time, *RSI* Reflux Symptom Index, *RSS 12* Reflux Symptom Score 12, *RSA -* Reflux Sign Assessment

## Discussion

LPR treatment of patients with chronic cough is not standardized, and PPIs are frequently chosen as first-line therapy, although their effect is often insufficient, even at maximum doses. In patients with only LPR symptoms, there is insufficient evidence to support the use of empirical PPI therapy [10]. Moreover, a recent randomized controlled trial revealed no significant difference between PPIs and placebo in terms of effectiveness for persistent throat symptoms [4]. This limitation stems from the fact that PPIs are primarily designed to reduce stomach acidity, rather than to prevent reflux episodes [11]. Moreover, long-term PPI use is linked to various health risks, including kidney disease, liver issues, cardiovascular complications, dementia, gastrointestinal infections, nutrient deficiencies, and neurological side effects [12].

Recently, there has been a shift toward studying the effects of dietary and lifestyle interventions as first-line treatment for LPR. For many patients with LPR, dietary measures such as a high-protein, alkaline, plant-based diet with low sugar content are sufficient treatment [2]. Smith et al. demonstrated a significant correlation between high calorie and fat intake and the intensity of cough symptoms [13]. Similarly, Yeakel et al. found that dietary and lifestyle changes yielded subjective improvement of chronic cough in 60% of patients with LPR [14].

The pathophysiology of LPR involves the retrograde movement of gastric contents into the larynx and pharynx, which causes inflammation and irritation, and can lead to chronic cough among other symptoms. Kikuchi et al. reported the presence of LPR and micro-aspiration of gastric contents into the larynx in a patient with chronic cough [9]. Dietary modifications that minimize acidic and reflux-inducing foods play a crucial role in reducing the frequency and intensity of reflux episodes. However, patient adherence to dietary recommendations is essential for the long-term success of non-pharmacological treatment. Patient noncompliance can result in treatment failure [15]. Therefore, our study also focused on thorough patient education about the dietary regimen. We provided detailed list of foods and drinks that are unsuitable for an anti-reflux diet, as well as those that are suitable. Patients also received a detailed dietary plan in written form, and dedicated sufficient time to patient education.

MII-pH impedance monitoring is currently the gold standard for LPR diagnosis. However, this procedure is often quite uncomfortable for patients, which sometimes results in incomplete testing. The results of our present study indicate that in patients with mild chronic cough and symptoms or signs suggestive of LPR, anti-reflux dietary measures can be implemented immediately, without the need for uncomfortable and semi-invasive HEMII-pH testing.

Our results demonstrated that an anti-reflux diet alone was effective for patients with mild chronic cough, with improvement observed in over 80% of cases. This conclusion is consistent with growing evidence emphasizing dietary and lifestyle modifications as key first-line treatments for LPR management, especially in patients with mild symptoms. Interestingly, our results showed greater improvement with the anti-reflux diet among patients with mild cough, higher initial RSI scores, and a higher number of reflux episodes on HEMII-pH. This suggests that if patients with mild symptoms do not show improvement after following an anti-reflux diet, the addition of PPIs and alginates is unlikely to lead to further improvement. It is likely that in this patient group, the cough is caused by other factors.

## Conclusion

An anti-reflux diet alone was an effective and sufficient treatment for patients with mild chronic cough caused by LPR. In clinical practice, these findings may encourage a shift away from the empirical use of PPIs and semi-invasive diagnostic methods for patients with mild cough symptoms.
